# Heterologous Prime-Boost Immunization with DNA Vaccine and Modified Recombinant Proteins Enhances Immune Response against *Trueperella pyogenes* in Mice

**DOI:** 10.3390/vaccines10060839

**Published:** 2022-05-25

**Authors:** Ting Huang, Kelei Zhao, Xuhao Song, Tao Song, Xinrong Wang, Xiuyue Zhang, Bisong Yue, Yiwen Chu

**Affiliations:** 1 Antibiotics Research and Re-Evaluation Key Laboratory of Sichuan Province, School of Pharmacy, Chengdu University, Chengdu 610052, China; zkl5228@163.com (K.Z.); songtao@cdu.edu.cn (T.S.); wang1593@sina.com (X.W.); 2 Key Laboratory of Southwest China Wildlife Resources (Ministry of Education), China West Normal University, Nanchong 637002, China; songxuhao1991@163.com; 3 Key Laboratory of Bio-Resources and Eco-Environment (Ministry of Education), College of Life Sciences, Sichuan University, Chengdu 610041, China; zhangxy317@126.com (X.Z.); bsyue@scu.edu.cn (B.Y.)

**Keywords:** *Trueperella pyogenes*, DNA vaccine, recombinant proteins, heterologous prime-boost, pyolysin

## Abstract

*Trueperella pyogenes* (*T. pyogenes*) is a crucial opportunistic pathogen normally causing mastitis, abscesses and pneumonia in economically important ruminants. Although only one commercial vaccine of *T. pyogenes* is currently obtainable, its immunoprotective effect is limited. Pyolysin (PLO) is the most predominant virulence factor highly expressed in *T. pyogenes* and is an excellent target for the development of novel vaccines against *T. pyogenes*. In this study, we designed a heterologous prime-boost vaccination scheme combining a DNA vaccine pVAX1-PLO and a subunit vaccine His-PLO to maximize host responses in mice. Humoral and cellular immune responses and protective effects were evaluated in mice to compare the immunogenicity induced by different immunization schemes. Compared to the PBS-control group, in vivo immunization results showed that better immune responses of mice immunized with the pVAX1-PLO plasmids and His-PLO proteins were induced. The residual bacterial burdens from the liver and peritoneal fluid were remarkably decreased in the immunized mice compared with the PBS group. Notably, the heterologous prime-boost vaccination groups significantly enhanced host humoral and cellular immune responses and protected mice from different virulent *T. pyogenes* strains infection. Conclusively, this study provides a favorable strategy for the further development of next-generation vaccines against *T. pyogenes* infections.

## 1. Introduction

*Trueperella pyogenes* (*T. pyogenes*) is a well-recognized and ubiquitous Gram-positive pathogen causing severe mastitis, abscesses and pneumonia. This opportunistic bacterium can be isolated from domestic ruminants’ mucous membranes or skin, and leads to significant economic losses in the animal husbandry industry [1,2,3]. Moreover, *T. pyogenes* is considered to be an important pathogen of abscesses in endangered forest musk deer (*Moschus berezovskii*), which is now protected by Chinese legislation to prevent the decline of population numbers [4,5]. As a significant zoonotic pathogen, *T. pyogenes* can also infect humans and cause severe diseases, such as septicemia, pharyngitis, arthritis and various suppurative lesions [1,6].

Antibiotic therapies such as beta-lactams and tetracyclines are currently available to treat *T. pyogenes* infection, while the emergence of drug-resistant strains poses a serious challenge to veterinary practice. It is probably a threat to human health because of the mobile genetic elements in the bacterium and the selective pressure of antibiotics [7,8,9]. Vaccines, such as inactivated vaccines or protein vaccines, are another common approaches against *T. pyogenes* infection [10,11]. Although one commercial vaccine for *T. pyogenes* is available to date, based on an important meeting report in the World Organization for Animal Health (OIE), it has unsatisfactory results in terms of its immune protection [10]. We have previously shown that DNA vaccine co-immunized with IL-1β adjuvant enhanced hosts’ immune responses and protected mice from *T. pyogenes* infection [12]. However, a previous report showed that the immune responses induced by the constructed DNA vaccines are relatively low in large animals because the amount of DNA plasmids required for efficacious immunization is much greater [13]. Thus, developing a more effective vaccine strategy for treating *T. pyogenes* infection is essential. Recently, heterologous prime-boost immunization administration combined with DNA, subunit vaccines or adenovirus represents alternative and efficient approaches against many pathogens, such as bacteria, viruses and parasites [14,15,16,17]. This strategy is remarkably effective for augmenting both humoral and cellular immune responses, which comprises priming the host immune system against a distinct antigen and boosting antigen-specific responses induced by a target immunogen [18]. For instance, an interesting study showed that the DNA prime-protein boost tactic was efficacious in inducing a rapid increase in a specific antibody titer against infection and was critical for broadening the T-cell repertoire in T-cell-dependent antibody responses [17]. However, whether the heterologous prime-boost regimes could be applied to control *T. pyogenes* infection remains an open question.

In the present study, to enhance the immunogenicity of the DNA vaccine for preventing *T. pyogenes* infections, a heterologous prime-boost vaccination scheme was developed, which combined a DNA vaccine pVAX1-PLO and a subunit vaccine His-PLO to maximize humoral and cellular responses in a mouse model. The specific immune responses elicited by the different vaccination strategies in mice were evaluated. Additionally, the potential protection of the novel designed vaccine regime was determined by challenging with *T. pyogenes*. In conclusion, the interrelations between different immune attributes generated following vaccination provided better comprehension of the adaptive immune response against *T. pyogenes*.

## 2. Materials and Methods

### 2.1. Bacterial Strains, Plasmids and Growth Conditions

Two different virulent *T. pyogenes* strains, TP8 (moderately virulent) and TP7 (highly virulent) were preserved in the laboratory and were incubated on a blood agar plate at 37 °C, 5% CO_2_ [19,20]. *Escherichia coli* (*E. coli*) DH5α containing pVAX1-PLO plasmid and *E. coli* Rosetta (DE3) containing pET-28a-PLO plasmid were stored in the laboratory [12]. The pVAX1-PLO plasmid was extracted using Endo-Free Plasmid Maxi Kit following the manufacturer’s instructions (Omega, Norcross, GA, USA).

### 2.2. Optimization of Recombinant Protein Expression in E. coli Rosetta

Recombinant proteins were expressed and purified in an optimizing condition as described in a published report [12]. Briefly, the *E. coli* Rosetta containing pET-28a-PLO plasmid in LB liquid medium was shaken at 37 °C for 4 h until the optical density (OD600) value reached 0.4. This culture was supplemented with 0.4 mM isopropyl β-D-thiogalactoside (IPTG), and was shaken at 16 °C for 12 h to induce the production of modified recombinant protein His-PLO. The recombinant proteins His-PLO were separated in 16% (*w*/*v*) sodium dodecyl sulfate-polyacrylamide (SDS-PAGE) gels as described previously [21]. The cultured products were purified by HisTrap affinity column, following the manufacturer’s instructions (GE Healthcare, Madison, WI, USA).

### 2.3. Mice Models

All animal procedures were carefully performed in accordance with the Institutional Animal Care and Use Committee guidelines and permissions (Sichuan University). The outbred specific-pathogen-free female Kunming (KM) mice at 7 weeks of age were obtained from Dossy Experimental Animals (Chengdu, China) and were randomly divided into 5 different groups (15 mice/group). The 5 groups were as follows: PBS group, pVAX1-PLO plasmid group, modified His-PLO protein group, His-PLO protein prime-pVAX1-PLO plasmid boost group (P prime-D boost), and pVAX1-PLO plasmid prime-His-PLO protein boost group (D prime-P boost). The mice were anesthetized with ketamine and were primed with plasmids pVAX1-PLO (50 μg/mouse) or recombinant protein His-PLO (50 μg) as well as 50 μL PBS in the rectus femoris muscle. The mice were boosted with recombinant protein His-PLO or pVAX1-PLO on day 21 after prime immunization using the same vaccination protocols.

### 2.4. IgG Antibody Detection

The peripheral blood of mice was collected before vaccination and at 21 days post-immunization (dpi) and 42 dpi, respectively. The serum from blood was obtained at 37 °C for 1 h and centrifuged at 1000 rpm for 3 min. The potency of PLO-specific antibodies was determined to evaluate the host humoral immune response via ELISA assay as described in a published report [12]. Briefly, the purified His-PLO protein was diluted to 10 μg/mL in ELISA coating buffer (Solarbio Life Science, Beijing, China) and coated in the 96-well ELISA plate overnight at 4 °C. Sera from each group (1:500 in PBST) were used as the primary antibodies, and the horseradish peroxidase (HRP)-labeled goat anti-mouse IgG (1:3000 dilution in PBST) was used as a secondary antibody. Absorbance at 492 nm was recorded on a microplate reader. To determine whether this prime-boost vaccine induced Th1-type or Th2-type immune response, the serum antibody production of IgG1 or IgG2a subtype was detected at different time points by ELISA assay as descried above.

### 2.5. MTT Assay

Proliferation of the lymphocytes was detected using an MTT assay (3-[4,5-dimethylthiazol-2-yl]-2,5-diphenyl tetrazolium bromide) as previously described [22]. Briefly, the lymphocytes in the spleen were isolated using a lymphocyte separation medium kit (Solarbio Life Science). Cells (1 × 10^7^ cells/mL, 50 μL/well) were seeded in a 96-well plate with the same volume of medium containing 20 μg/mL of purified His-PLO protein as a stimulus. A total of 50 μL DMEM was used as a negative control. The cells were cultivated for 48–72 h at 37 °C and then MTT reagent was added to the 96-well plate and incubated for 4 h in a humidified chamber. The formazan dye was detected by measuring the absorbance at 490 nm on a microplate reader. All treatments were performed in triplicate. 

### 2.6. Cytokine Profiling

The cytokine profiles (IFN-γ, IL-2 and IL-4) in the supernatant of the spleen lymphocytes were determined using the different ELISA kits (Solarbio Life Science) following the manufacturer’s instructions. The levels of different cytokines were calculated according to the corresponding standard curves as previously described [23].

### 2.7. Challenge Experiments

The different vaccination groups were intraperitoneally injected with 3.7 × 10^8^ CFU TP7 strain or TP8 strain at three weeks after the second immunization. The bacterial burdens in the liver and peritoneal fluid (PF) were determined at day 5 post-infection and were counted on a brain heart infusion agar plate. Additionally, the mortality of the challenged mice was examined for 30 days as previously described [19]. 

### 2.8. Histological Analysis

The liver samples of different groups of immunized mice were harvested aseptically after the necropsy and fixed in 10% formalin solution. Subsequently, the paraffin-embedded tissue sections were obtained using a rotary microtome and hematoxylin-eosin staining as previously described [5]. All sections of liver samples were examined by light microscopy. Triplicates were performed for each control and sample.

### 2.9. Statistical Analysis

All the data were analyzed (one-way analysis of variance (ANOVA), Tukey–Kramer post hoc test, a chi-square test with Yates’ correction) using GraphPad Prism 5.0 (GraphPad Software, La Jolla, CA, USA). A probability value of *p* < 0.05 was considered significant.

## 3. Results

### 3.1. Characterization of Recombinant His-PLO Protein

**Figure 1:**
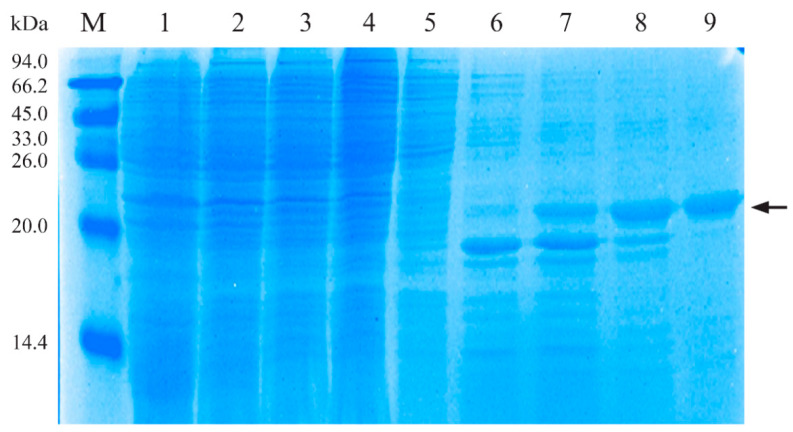
Characterization of recombinant His-PLO. Purification of His-PLO protein was detected with Coomassie-stained SDS-PAGE gels. M, protein marker; 1, total bacterial proteins not induced; 2, total bacterial proteins induced for 12 h; 3, supernatant from crushed cells; 4, flow-through solutions; 5–9, purified recombinant His-PLO protein by different concentrations of imidazole solutions (25 mM, 50 mM, 75 mM, 100 mM, 150 mM). Arrow on the right indicates the specific His-PLO band detected, which corresponds to the 21 kDa protein.

As shown in Figure 1, the plasmid pET-28a-PLO in *E. coli* Rosetta (DE3) was induced under the optimizing condition, and the putative truncated peptide of PLO was expressed at about 21 kDa. Recombinant His-PLO protein was purified and this band corresponds to the expected molecular size of the specific antigen.

### 3.2. Prime-Boost Regimens Elicited Higher Antibody Production

**Figure 2:**
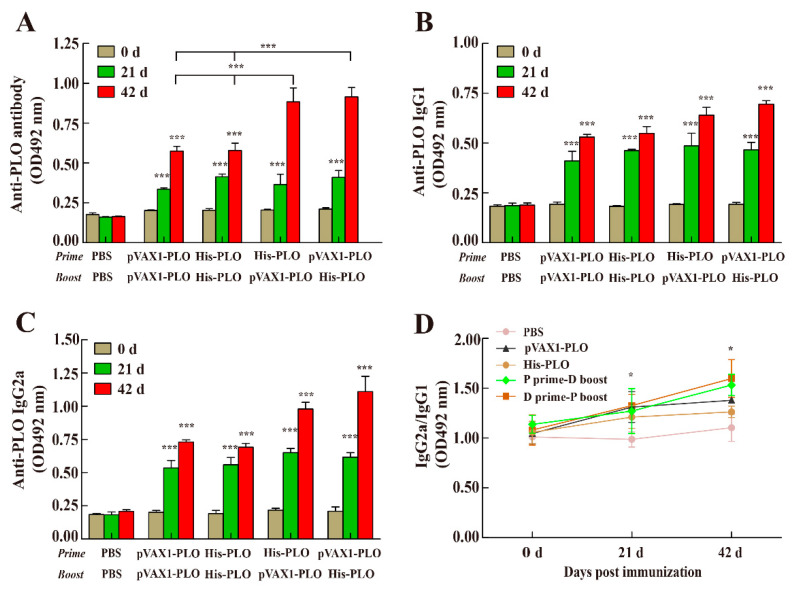
Antibody responses induced by the prime-boost regimens. Mice were immunized with plasmid pVAX1-PLO or recombinant His-PLO protein and boosted once at day 21 after primary immunization. Sera samples were collected at the indicated time points. The anti-PLO IgG (**A**), IgG1 (**B**) and IgG2a (**C**) levels were detected by indirect ELISA. (**D**) Dynamic changes of sera IgG2a/IgG1 against PLO in immunized mice. PBS was used as a control. Data represented means ± SEM of three independent experiments. * *p* < 0.05, *** *p* < 0.001.

To investigate whether the immunogenicity of prime-boost regimens was higher than that elicited by a monovalent DNA vaccine or protein vaccine, antibody responses to the target antigen were determined using ELISA. The results showed that vaccination with the pVAX1-PLO DNA vaccine or His-PLO protein significantly increased the production of PLO-specific antibodies in immunized mice at 21 dpi (Figure 2A). The specific antibody response was continually increased after the boost at 42 dpi. In addition, the antibody production in heterologous immunization groups was higher than the single DNA vaccine or single protein vaccine group at 42 dpi, while the antibody response in PBS groups was not statistically different. As shown in Figure 2B,C, the serum antibody production of the IgG1 and IgG2a subtypes was determined at indicated time points in immunized mice (Figure 2B,C). Moreover, the antibody production of IgG2a was greater than the IgG1 subtype, Figure 2D (a ratio of OD values), indicating that prime-boost regimens mainly induced a Th1-type immune response.

### 3.3. Prime-Boost Regimens Induced Higher Cellular Immune Response

**Figure 3:**
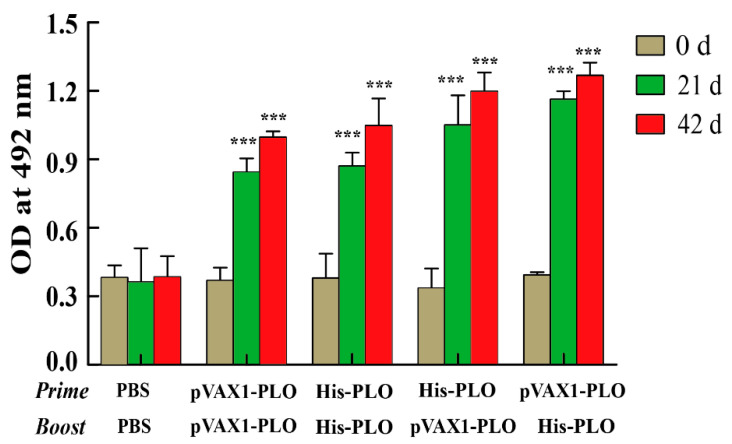
Proliferation of lymphocytes in the spleens of immunized mice. Spleens were collected from the immunized mice at indicated time point. PBS was used as a control. The lymphocytes from the spleen of mice were separated and their proliferation was determined by MTT assay. The purified recombinant His-PLO protein was used as stimuli. Absorbance was detected at 490 nm. Data represented means ± SEM of three independent experiments. *** *p* < 0.001.

**Figure 4:**
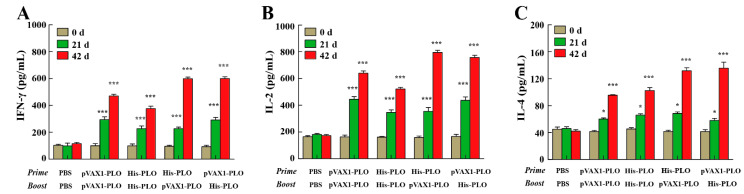
Levels of secreted cytokines from the suspension of the spleen lymphocytes in immunized mice. The spleens were collected at the indicated time points. The levels of IFN-γ (**A**), IL-2 (**B**) and IL-4 (**C**) from the suspension of the spleen lymphocytes were detected by ELISA assays. Data represented means ± SEM of three independent experiments. * *p* < 0.05, *** *p* < 0.001.

The changes in lymphocytes and the levels of cytokines induced by prime-boost regimens were detected to investigate the cellular immune response in immunized mice. The results showed that the values from the pVAX1-PLO DNA vaccine group or His-PLO protein group were increased by the MTT assay at 21 dpi. However, such effects were not observed in the PBS groups (Figure 3). The total lymphocytes continually proliferated after the boost at 42 dpi, and the prime-boost group induced more lymphocytes compared with the pVAX1-PLO group or His-PLO group alone in the spleen, as shown in Figure 3. As determined by ELISA, we found that the levels of IFN-γ, IL-2 and IL-4 in the supernatant of the spleen lymphocytes were significantly augmented in the pVAX1-PLO group or His-PLO proteins group alone at 21 dpi and were promoted by prime-boost regimens at 42 dpi (Figure 4A–C). No significant changes in cytokines were detected in the supernatant of the spleen lymphocytes of the PBS groups.

### 3.4. Prime-Boost Immunization Protected Mice against T. pyogenes Infection

**Figure 5:**
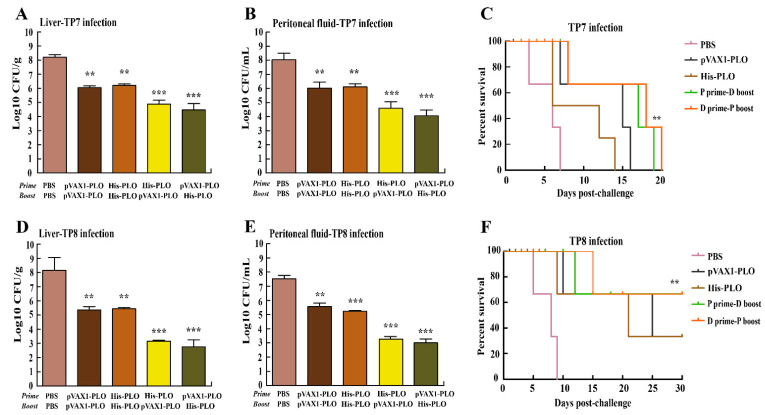
The bacterial burdens and survival rate of mice challenged with *T. pyogenes*. The immunized mice were intraperitoneally challenged at day 43 with *T. pyogenes* TP7 or TP8 strain to determine bacterial burdens from liver (**A**,**D**) and peritoneal fluid (**B**,**E**) samples. Survival rate was monitored for the subsequent 30 days (**C**,**F**). PBS was used as a control. ** *p* < 0.01 and *** *p* < 0.001.

**Figure 6:**
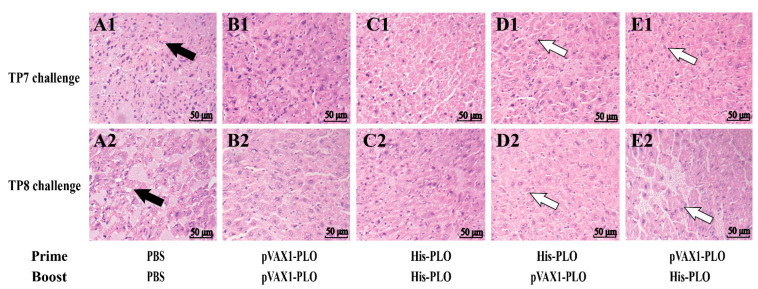
Histological analysis of immunized mice challenged with *T. pyogenes*. The immunized mice were intraperitoneally challenged at day 43 with *T. pyogenes* TP7 (**A1**–**E1**) or *T. pyogenes* TP8 (**A2**–**E2**), respectively. The livers of immunized mice were collected to assess the histology alteration. PBS was used as a control. Abnormal hepatocytes and cellular debris in the PBS group are indicated by the black arrows. The hepatocytes with fewer pathological changes in the prime-boost immunized groups are indicated by the white arrows.

To further investigate the protective ability of prime-boost-immunization-raised immunity to clean *T. pyogenes*, the bacterial burdens in the liver or PF were detected after challenge with *T. pyogenes*. As shown in Figure 5, in the mice that were challenged with TP7 or TP8, abundant bacteria CFU from the liver and PF of mice were determined in the PBS group. Additionally, prime-boost vaccination promoted significant elimination of bacteria compared with the DNA vaccine-treated or protein-treated group in the liver (Figure 5A,D) and PF (Figure 5B,E). As demonstrated in Figure 5C,F, PBS-treated mice died on day 7 or day 8 following *T. pyogenes* infection. Nevertheless, the mice immunized with single DNA vaccine or His-PLO protein showed a higher survival rate than the control group (Figure 5C). Importantly, most mice displayed few disease symptoms due to prime-boost immunization and higher survival against TP8 infection at 30 dpi (Figure 5F). Furthermore, the livers of PBS-treated mice showed a number of impaired hepatocytes and much cellular debris located in the necrotic center (Figure 6(A1,A2)). Whereas the mice immunized with single DNA vaccine or His-PLO protein significantly reduced the invading bacteria in the livers following *T. pyogenes* infection (Figure 6(B1–C2)) and the liver tissue of mice showed few pathological changes due to prime-boost immunization (Figure 6(D1–E2)).

## 4. Discussion

As an important zoonotic pathogen, *T. pyogenes* is considered to cause miscellaneous pyogenic infections in most ruminant animals [1,24]. The multifaceted pathogenicity and the diverse virulence factors in *T. pyogenes* are major impediments to developing effective universal vaccines. A previous study showed that vaccination experiments using inactivated cells were ineffective in preventing ruminants from contracting *T. pyogenes* infections [25]. Although pyolysin (PLO) was the predominant virulent factor in *T. pyogenes*, vaccination with native or recombinant PLO protein exhibited fewer immunoprotective effects for immunized mice against the challenge of *T. pyogenes* [10,26]. In the present study, we developed a novel vaccine strategy against *T. pyogenes* using rationally designed immunogens and heterologous prime-boost vaccination platforms to enhance host immune responses at the humoral and cellular levels. Notably, the heterologous prime/boost vaccination induced stronger humoral immune responses and cellular immune responses and provided significant protective effects against *T. pyogenes* challenge.

As mentioned in our previous report, the specific antibody responses were the main index of humoral immune responses since the antibody serves a critical function in protecting the host from bacterial infections [12]. Analyses of IgG antibody responses showed that immunization with only DNA vaccines or a protein vaccine elicited higher antibody responses than the control group, similar to the observations on the designed DNA constructs or recombinant proteins against other severe diseases [10,15,27]. Interestingly, a significant increase in the antibody response in groups boosted with DNA vaccines or recombinant proteins was detected at 42 dpi compared to other groups, suggesting that the heterologous prime-boost approach is highly effective in producing rapidly specific antibody responses against *T. pyogenes*. This aligns with the findings on DNA prime-protein boost vaccinations against anthrax caused by *Bacillus anthracis* [17]. Therefore, our study proved the induction of humoral immune responses by the heterologous prime-boost regimes in mice, compared to all the immunized groups.

A noteworthy observation demonstrated that the cellular immune responses played an important role in the host response to *T. pyogenes* infection [19]. Accordingly, an MTT assay was deployed to detect the proliferation of lymphocytes. We found that the heterologous prime-boost regime induced higher lymphocyte proliferation in immunized mice than DNA vaccines or protein vaccines alone. As the secreted level of cytokines was considered to be the most real-time reflector of cellular immune regulation in the host [28,29], a cytokine profiling assay was subsequently used to detect the release of IL-2, IFN-γ and IL-4. Concentrations of the target cytokines were remarkably higher in the vaccinated groups compared to the control group, suggesting that both Th1 and Th2 types of immune response were induced by the vaccination strategy, as mentioned above. Interestingly, the heterologous prime-boost groups experienced a significantly enhanced release of IFN-γ, IL-2 and IL-4 compared to the other groups. These crucial interactions show the immunoprotective effect of the host defense against *T. pyogenes* infections and the development of specific antibacterial immunity. In line with these findings, a previous study also demonstrated that DNA prime-protein boost vaccination promoted lymphocytes maturation by inducing IFNs and further enhancing host cellular immunity [30]. Notably, both heterologous vaccination regimes showed similar effects for considerably enhancing the number of the lymphocytes and secreted cytokines, revealing that PLO functioned as an important priming agent and an effective boosting platform. The present study demonstrated that the heterologous prime-boost regimes induced higher levels of lymphocyte proliferation and cytokine secretion in immunized mice than in the control group, which might provide efficacious protection from *T. pyogenes* infections.

To further explore the immunoprotective effects of the heterologous prime-boost regimes against *T. pyogenes* challenge, the bacterial burdens and survival rates were detected in the immunized mice. Similar to previous work [19,31], we found that large amounts of bacteria were detected in the livers and PF in the PBS group. In contrast, the mice boosted with the His-PLO protein or pVAX1-PLO DNA vaccine showed less bacteria in the livers and PF, indicating that most of the bacteria were eliminated from the immunized mice. A significantly higher cumulative survival rate of mice was observed in different immunized groups compared to the control group. The maximum survival rate was shown by the heterologous prime-boost vaccination approach. Notably, the mice infected with TP7 eventually succumbed to the infection, suggesting that the rest of TP7 might be persistent in the mice and cause death in late infection. Nevertheless, the heterologous prime-boost vaccinations significantly protected most mice from TP8 infection. Altogether, our findings further confirmed that this novel immunization approach improved host immune responses and provided global immunoprotective effects against *T. pyogenes* infections.

## 5. Conclusions

In conclusion, using a mouse model, our study clearly demonstrated that the pVAX1-PLO DNA vaccine or modified His-PLO protein vaccine elicited humoral and cellular immune responses and provided immunoprotective effects against *T. pyogenes* infections. Immunization with a heterologous prime-boost strategy generated higher protective immunity against *T. pyogenes*. Importantly, these findings indicate that the DNA prime-protein boost or protein prime-DNA boost regime based on PLO is an effective strategy for developing a next-generation *T. pyogenes* vaccine. A more comprehensive study using a heterologous vaccination strategy for large ruminants will broaden our understanding of combating *T. pyogenes* diseases.

## Data Availability

Not applicable.
